# Quantification of carotid plaque composition with a multi-contrast atherosclerosis characterization (MATCH) MRI sequence

**DOI:** 10.3389/fcvm.2023.1227495

**Published:** 2023-08-23

**Authors:** Mohamed Kassem, Kelly P. H. Nies, Ellen Boswijk, Jochem van der Pol, Mueez Aizaz, Marion J. J. Gijbels, Debiao Li, Jan Bucerius, Werner H. Mess, Joachim E. Wildberger, Robert J. van Oostenbrugge, Rik P. M. Moonen, Zhaoyang Fan, M. Eline Kooi

**Affiliations:** ^1^ Cardiovascular Research Institute Maastricht (CARIM), Maastricht University Medical Center, Maastricht, Netherlands; ^2^ Department of Radiology and Nuclear Medicine, Maastricht University Medical Center, Maastricht, Netherlands; ^3^ Department of Rehabilitation Medicine, Amsterdam University Medical Center, Location VUmc, Amsterdam, Netherlands; ^4^ Department of Pathology, Cardiovascular Research Institute Maastricht, Maastricht University Medical Center, Maastricht, Netherlands; ^5^ Department of Medical Biochemistry, Experimental Vascular Biology, Amsterdam Cardiovascular Sciences, Amsterdam Infection and Immunity, Amsterdam UMC, Amsterdam, Netherlands; ^6^ Biomedical Imaging Research Institute, Cedars-Sinai Medical Center, Los Angeles, CA, United States; ^7^ Department of Nuclear Medicine, Georg-August University Göttingen, Universitätsmedizin Göttingen, Göttingen, Germany; ^8^ Department of Clinical Neurophysiology, Maastricht University Medical Center, Maastricht, Netherlands; ^9^ Department of Neurology, Maastricht University Medical Center, Maastricht, Netherlands; ^10^ Department of Radiology, University of Southern California, Los Angeles, CA, United States

**Keywords:** magnetic resonance imaging, atherosclerotic plaque, carotid arteries, stroke, MATCH, MATCH MRI sequence for quantifying carotid plaque composition

## Abstract

**Background and purpose:**

Carotid atherosclerotic plaques with a large lipid-rich necrotic core (LRNC), intraplaque hemorrhage (IPH), and a thin or ruptured fibrous cap are associated with increased stroke risk. Multi-sequence MRI can be used to quantify carotid atherosclerotic plaque composition. Yet, its clinical implementation is hampered by long scan times and image misregistration. Multi-contrast atherosclerosis characterization (MATCH) overcomes these limitations. This study aims to compare the quantification of plaque composition with MATCH and multi-sequence MRI.

**Methods:**

MATCH and multi-sequence MRI were used to image 54 carotid arteries of 27 symptomatic patients with ≥2 mm carotid plaque on a 3.0 T MRI scanner. The following sequence parameters for MATCH were used: repetition time/echo time (TR/TE), 10.1/4.35 ms; field of view, 160 mm × 160 mm × 2 mm; matrix size, 256 × 256; acquired in-plane resolution, 0.63 mm^2^× 0.63 mm^2^; number of slices, 18; and flip angles, 8°, 5°, and 10°. Multi-sequence MRI (black-blood pre- and post-contrast T1-weighted, time of flight, and magnetization prepared rapid acquisition gradient echo; acquired in-plane resolution: 0.63 mm^2^ × 0.63 mm^2^) was acquired according to consensus recommendations, and image quality was scored (5-point scale). The interobserver agreement in plaque composition quantification was assessed by the intraclass correlation coefficient (ICC). The sensitivity and specificity of MATCH in identifying plaque composition were calculated using multi-sequence MRI as a reference standard.

**Results:**

A significantly lower image quality of MATCH compared to that of multi-sequence MRI was observed (*p* < 0.05). The scan time for MATCH was shorter (7 vs. 40 min). Interobserver agreement in quantifying plaque composition on MATCH images was good to excellent (ICC ≥ 0.77) except for the total volume of calcifications and fibrous tissue that showed moderate agreement (ICC ≥ 0.61). The sensitivity and specificity of detecting plaque components on MATCH were ≥89% and ≥91% for IPH, ≥81% and 85% for LRNC, and ≥71% and ≥32% for calcifications, respectively. Overall, good-to-excellent agreement (ICC ≥ 0.76) of quantifying plaque components on MATCH with multi-sequence MRI as the reference standard was observed except for calcifications (ICC = 0.37–0.38) and fibrous tissue (ICC = 0.59–0.70).

**Discussion and conclusion:**

MATCH images can be used to quantify plaque components such as LRNC and IPH but not for calcifications. Although MATCH images showed a lower mean image quality score, short scan time and inherent co-registration are significant advantages.

## 1. Introduction

Stroke is the second global cause of both disability and mortality (1). Approximately 85% of all strokes are classified as ischemic strokes (2), and approximately 20% of ischemic strokes are linked to carotid atherosclerosis (3). At present, the degree of carotid stenosis is essential in determining the type of (secondary) stroke prevention treatment. Ischemic stroke related to carotid stenosis is mainly caused by embolization occurring after plaque rupture rather than by low perfusion resulting from the stenotic plaque (4–6). A vulnerable plaque is a plaque that is more susceptible to rupture and distal embolization. The morphological features of a vulnerable plaque include the presence of a large lipid-rich necrotic core (LRNC), intraplaque hemorrhage (IPH), and a thin or ruptured fibrous cap (7–9). It has been demonstrated that these features are strongly associated with an increased risk of (recurrent) stroke (10–12).

In the last two decades, magnetic resonance imaging (MRI) has emerged as the preferred imaging modality for visualizing and assessing plaque composition (9, 13, 14). By employing a combination of different MRI pulse sequences, especially pre- and post-contrast T1-weighted (T1w) turbo spin echo (TSE), magnetization prepared rapid acquisition gradient echo (MPRAGE), and time of flight (TOF) MRI, various plaque components can be distinguished (15). Recently, expert consensus recommendations on using the abovementioned multi-sequence carotid vessel wall imaging protocol have been published (15, 16). However, multi-sequence carotid MRI still has some limitations. Limited slice resolution associated with 2D sequences, long acquisition time, and image misregistration due to patient motion between scans may hamper clinical implementation.

A few years ago, a new pulse sequence, i.e., multi-contrast atherosclerosis characterization (MATCH), was introduced (17). MATCH employs a 3D segmented spoiled gradient echo (GRE) readout to acquire data with three different contrast weightings, including hyper-T1w, gray-blood, and T2-weighted (T2w), following a non-selective inversion pulse and various inversion recovery times. On the hyper-T1w and T2w images, luminal blood signals are suppressed using flow-sensitive dephasing (FSD) magnetization preparation (18). IPH and calcifications can be delineated on the black-blood hyper-T1w and gray-blood images, respectively. The T2w images provide information on overall plaque morphology and the presence of LRNC (17). In addition to the unique tissue contrast weightings available in MATCH, all three image sets are simultaneously acquired in a 5-min scan and therefore are inherently co-registered and more immune to patient intolerance than conventional multi-sequence protocols. In a study conducted on a small group of six patients, good agreement between MATCH and multi-sequence MRI was demonstrated in detecting plaque components (17). However, this study had a few limitations, including a small sample size and no quantitative analysis of the plaque components. Later, in a study conducted on a large group of 46 patients, MATCH and multi-sequence MRI showed similar performance in detecting plaque components (19). An essential limitation of both studies was the absence of a dedicated sequence for detecting IPH or LRNC in the multi-sequence protocol. We used a dedicated sequence for the identification of IPH (MPRAGE) and LRNC (pre- and post-contrast T1w TSE images) as part of the multi-sequence protocol in the current study, as recommended in the consensus paper (16).

Therefore, the current study aims to compare the diagnostic performance of MATCH with multi-sequence MRI, including a dedicated sequence for IPH and LRNC to quantify carotid plaque composition.

## 2. Materials and methods

### 2.1. Study population

Baseline carotid MRI data between 2018 and 2022 were derived from three different prospective studies in our institution. These studies were registered at ClinicalTrial.gov (NCT03291093, NCT02640313, and NCT04569006). The common objective of these previous trials was to evaluate novel PET tracers and novel MRI sequences. Our institutional review board approved all three studies. A signed informed consent form was obtained from each patient. The inclusion criterion was the presence of a carotid plaque of at least 2 mm in the ipsilateral carotid artery on either duplex ultrasonography or computed tomography angiography (CTA). Both symptomatic patients, who had experienced a TIA, stroke, or retinal ischemia, and asymptomatic patients were eligible for inclusion. Only patients who underwent MATCH and multi-sequence MRI were eligible for inclusion in our analysis.

### 2.2. MRI protocol

All examinations were performed on a 3.0 T hybrid integrated PET-MRI scanner (Biograph mMR, Siemens Healthineers). MATCH and multi-sequence MRI were combined in one exam. A four-channel special-purpose coil (Siemens Healthineers) was used to image the carotid bifurcation, allowing submillimeter-resolution imaging. The scan parameters for MATCH were as follows: repetition time/echo time (TR/TE), 10.10/4.35 ms; field of view (FOV), 160 mm × 160 mm; acquired matrix size, 256 × 256; in-plane acquired resolution, 0.63 mm^2^ × 0.63 mm^2^; slice thickness: 2 mm; flip angles, 8°, 5°, and 10°; number of slices, 18; and bandwidth, 130 Hz/pixel. The multi-sequence MRI protocol acquired 14 adjoining transverse 2 mm slices covering the entire plaque. The parameters of multi-sequence MRI and MATCH are listed in Table 1. A 0.1 mmol/kg dose of gadolinium-based contrast medium (Gadovist, Bayer AG) was used for post-contrast T1w imaging with a delay of 6 min post-injection.

**Table 1:** MATCH and multi-sequence carotid MRI protocol.

Pulse sequence	Pre-/post-contrast T1w TSE	TOF FFE	MPRAGE	MATCH
Acquisition plane	Transversal	Transversal	Transversal	Transversal
Acquisition time (min:s)	5:14 × 2	2:47	3:25	4:44
Mean scan time (min:s)	39:31			7:25
Image mode	2D	3D	3D	3D
TR (ms)	800	20	13.2	10.1
TE (ms)	10	3.6	6.5	4.35
TI (ms)	683	n/a	500	450, 1,100, 3,600
Shot interval (ms)	n/a	n/a	800	4,239
Flip angle (°)	90	20	15	8,5,10
No. of slices	14	14	14	18
Slice thickness (mm)	2	1	2	2
FOV (mm)	160 × 160	160 × 160	160 × 160	160 × 160
Acquisition matrix	256 × 256	256 × 256	256 × 256	256 × 256
Acquired voxel size (mm)	0.63 × 0.63 × 2.0	0.63 × 0.63 × 1.0	0.63 × 0.63 × 2.0	0.63 × 0.63 × 2.0
Reconstructed voxel size (mm)	0.31 × 0.31 × 2.0	0.31 × 0.31 × 1.0	0.31 × 0.31 × 1.0	0.31 × 0.31 × 2.0
Echo train length	10	n/a	44	53
GRAPPA acceleration factor	n/a	n/a	n/a	2
No. of signal averages	1	1	1	1
Fat suppression	SPAIR	no	water excitation	water excitation

FFE, fast field echo; TI, inversion time; n/a, not applicable; SPAIR, spectral attenuated inversion recovery.

### 2.3. Image analysis

All image datasets were anonymized and processed using the dedicated software (VesselMass, Department of Radiology, Leiden University Medical Center). Two trained observers (MK and KN) with 1–3 years of experience reviewed the MR images independent of the clinical data and each other. The multi-sequence MRI and MATCH images were evaluated blindly from the delineations and the scores of the other MRI method with a time interval of at least 1 month.

The observers manually co-registered the images of the different contrast weightings of the multi-sequence protocol in-plane (*x*-direction and *y*-direction) and out-of-plane (*z*-direction) using the same dedicated software package (VesselMass). The time required for manual co-registration was included in the overall image analysis time. In the case of MATCH images, co-registration was inherent and did not require additional manual adjustments. Multi-sequence and MATCH images were assessed based on the previously validated criteria (17, 20). (1) The inner and outer vessel wall of the plaque was delineated on the pre-contrast T1w TSE images (multi-sequence protocol) or T2w images (MATCH protocol). (2) The LRNC was defined as a region within the plaque that exhibits no contrast enhancement on the post-contrast T1w images (multi-sequence protocol) or a hypo-intense region within the plaque on T2w images and isointense on hyper-T1w images (MATCH protocol). (3) IPH was characterized as a hyper-intense signal in the bulk of the plaque compared to surrounding muscle tissue. For the multi-sequence protocol and MATCH protocol, the MPRAGE images and hyper-T1-weighted images were used, respectively. (4) For the multi-sequence protocol, calcifications were identified as areas with a hypo-intense signal relative to the sternocleidomastoid muscle on at least two different MRI weightings. Juxtaluminal calcifications can be obscured on the dark blood MRI weightings; thus, the TOF images are used to identify juxtaluminal calcifications. For the MATCH protocol, calcifications were identified as a hypo-intense signal on the gray-blood images. The quantification of the total fibrous tissue involved subtracting the combined volume of the LRNC (including IPH) and calcifications from the overall volume of the vessel wall (21). The normalized wall index (NWI) was defined as wall area/(lumen area + wall area), and the wall area was defined as the area between the lumen and outer wall (22). The percent wall volume (PWV) is calculated by dividing the volume of the vessel wall by the total volume of the carotid artery segment and then multiplying this number by 100 to express it as a percentage (23). The scan time (acquisition time and planning) and the image analysis time (co-registration time and time to delineate the vessel wall and plaque components) were recorded for MATCH and multi-sequence MRI.

A region of interest (ROI) was drawn in the sternocleidomastoid muscle at the level of the carotid bifurcation. The signal-to-noise ratio (SNR) was calculated as the ratio between the mean signal intensity and the standard deviation (SD) of this ROI. The contrast-to-noise ratio (CNR) of IPH was calculated for MPRAGE and MATCH as the difference in mean signal intensity of IPH and muscle tissue divided by the noise of muscle tissue (SD) (24). The image quality was scored on a 5-point scale on a slice-by-slice basis: 1 for low image quality and 5 for excellent image quality (20).

### 2.4. Histological analysis

In one patient who underwent carotid endarterectomy as part of his/her routine clinical care 1 day after the MRI examination, the MRI findings were compared to histology. The specimen was collected directly after carotid endarterectomy. The specimen was cut into ∼3 mm slices, coded, and alternately frozen and stored at −80°C. The sample was fixated in a 4% paraformaldehyde phosphate buffered saline (PBS) solution for 18–48 h, decalcified using ethylenediaminetetraacetic acid (EDTA) for 4 h, and then embedded in paraffin. Cross-sectional sections of 4 mm were stained using hematoxylin and eosin (H&E). An experienced vascular pathologist macroscopically assessed the carotid plaque composition. All procedures conducted during the research adhered to the guidelines outlined in the Dutch Code of Conduct for Observational Research with Personal Data (2004) and Tissue.

### 2.5. Statistical analysis

Two-way mixed-effects model of intraclass correlation coefficients (ICCs) and Cohen's kappa test (*κ*) were used to assess the interobserver agreement for the quantification and identification of carotid plaque components on multi-sequence and MATCH images. The ICC is a numerical value ranging from 0 to 1. Values below 0.5 suggest poor agreement, values between 0.5 and 0.75 indicate moderate agreement, values between 0.75 and 0.9 represent good agreement, and any value above 0.9 indicates excellent agreement (25). Kappa values also range from 0 to 1, where values from 0 to 0.2 indicate slight; 0.21–0.4, fair; 0.41–0.60, moderate; 0.61–0.8, substantial; and 0.81, upward excellent agreement (26). The comparison between MATCH and multi-sequence protocol was as follows: hyper-T1w vs. MPRAGE, gray-blood vs. TOF, and T2w MATCH vs. post-contrast T1w TSE. Sensitivities and specificities of MATCH in identifying plaque components on an artery basis were calculated using the multi-sequence protocol as a reference standard. A paired *t*-test or Wilcoxon signed ranked test was used to evaluate the differences in vessel wall volume, NWI, and volumes of the various plaque components between the MATCH and multi-sequence protocol, as appropriate. In addition, the differences between the IPH, LRNC, calcifications, and NWI measurements on MATCH and multi-sequence images were plotted against the mean difference as Bland–Altman plots. Limits of agreement were calculated as mean difference ± 1.96 × standard deviation of difference. Statistical analyses were performed using IBM SPSS Statistics for Windows, Version 24.0 (IBM Corporation). Normally distributed continuous variables were presented as mean ± standard error. Otherwise, the median and interquartile range (IQR) was presented. *P* < 0.05 indicated statistical significance.

## 3. Results

**Figure 1:**
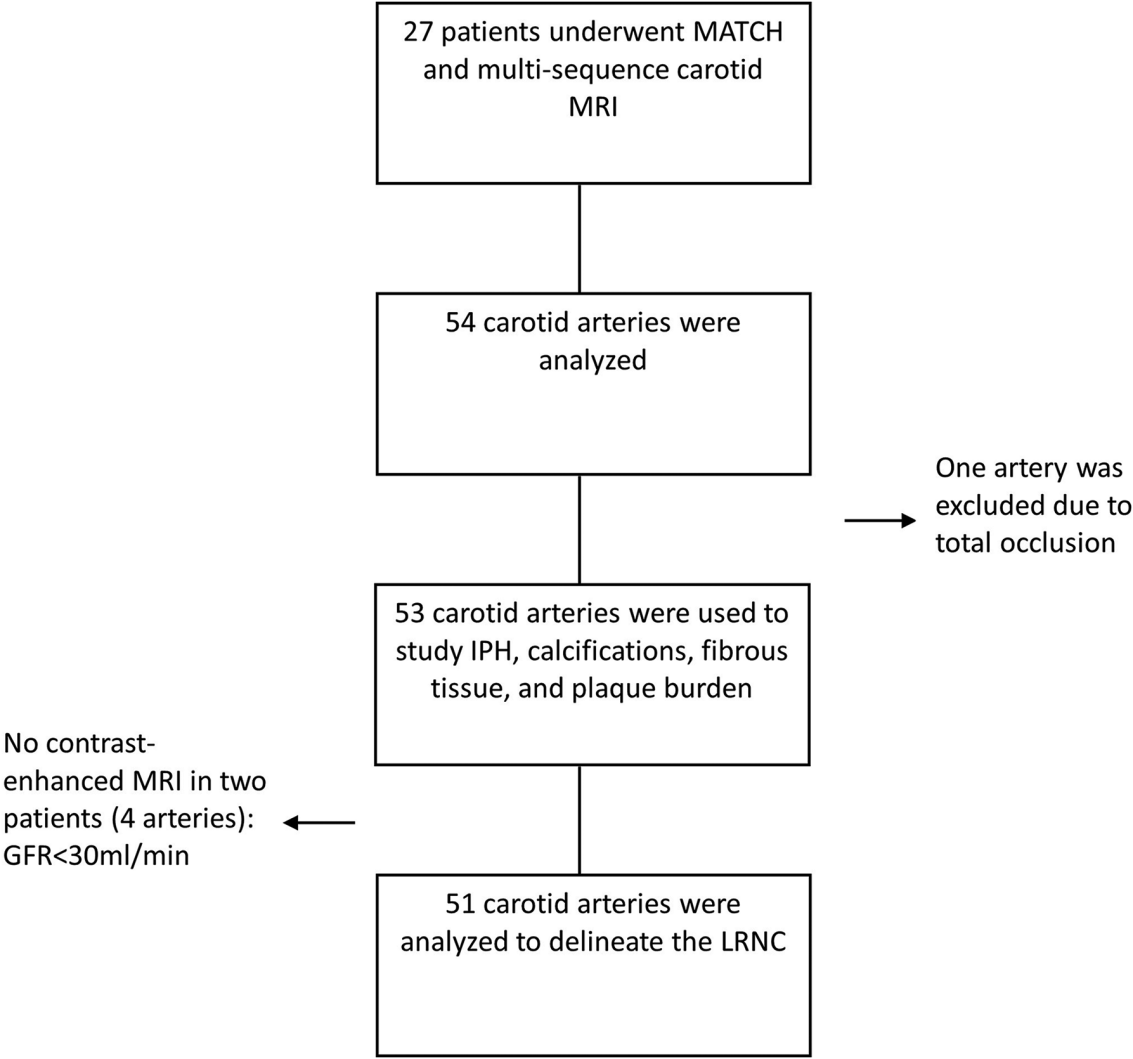
Flowchart of the study.

A total of 27 patients (54 carotids) underwent MATCH and multi-sequence carotid MRI, of which 21 were male. A flowchart of the patient inclusion is shown in Figure 1. One artery was excluded due to total occlusion. Two patients did not undergo contrast injection because of a low glomerular filtration rate (GFR) (<30 ml/min). The patients had a mean age of 70 ± 6.8 years. Patient characteristics are presented in Table 2. The mean MATCH and multi-sequence scan time was 7 min and 25 s and 39 min and 31 s, respectively. The mean time to delineate the vessel wall and the plaque components, including co-registration, for one artery on MATCH was shorter than that for the multi-sequence images (7:42 min ± 2:30 min and 13:24 ± 1:55 min, respectively; *p* < 0.05).

**Table 2:** Subject characteristics (*n* = 27; 53 arteries).

Characteristic	Value
Age, years; mean ± SD	70 ± 6.8
Sex, male; number (%)	21 (80%)
Stenosis degree (NASCET, %)	
<50%	37/54 (69%)
50%–69%	11/54 (20%)
70%–99%	5/54 (9%)
Time event to MRI (days), median (IQR)	9 (6–17)
Type of event, number (%)	
Stroke	13 (48%)
TIA	10 (37%)
Retinal ischemia	4 (15%)
Smoking status, number (%)	
Current	10 (37%)
Former smoker	8 (30%)
Never	9 (33%)
Hypertension; number (%)	15 (56%)
Hypercholesterolemia; number (%)	17 (63%)
Diabetes mellitus; number (%)	3 (11%)
Previous CVD and PAD; number (%)	6 (22%)
BMI (kg/m2); mean ± SD	25.9 ± 3.2

The data are presented as mean ± SD, median, IQR, or absolute numbers of patients (%).

NASCET, North American symptomatic carotid endarterectomy trial; CVD, cardiovascular disease; PAD, peripheral arterial disease; BMI, body mass index.

### 3.1. Image quality

A significantly lower image quality of hyper-T1w, gray-blood, and T2w MATCH images was found compared with MPRAGE, TOF, and post-contrast T1w TSE images [median score 1, IQR (1–1) vs. median score 4, IQR (3–4); 2, (2–3) vs. 4, (3–4); 3, (3–4) vs. 4, (4–4), respectively; *p* < 0.05]. A direct comparison between the MATCH and the equivalent multi-sequence images is presented in Table 3.

**Table 3:** Image quality comparison between MATCH and multi-sequence protocol.

Comparison	Median image quality	IQR	*p*-value	Mean SNR ± SE	*p*-value
Hyper-T1w vs. MPRAGE	1 vs. 4	(1–1) vs. (3–4)	*p* < 0.05	3.8 ± 0.3 vs. 12.8 ± 0.6	*p* < 0.001
Gray-blood vs. TOF	2 vs. 4	(2–3) vs. (3–4)	*p* < 0.05	9.2 ± 0.6 vs. 11.9 ± 0.7	*p* = 0.007
T2w MATCH vs. post-contrast T1w	3 vs. 4	(3–4) vs. (4–4)	*p* < 0.05	8.9 ± 0.6 vs. 12.5 ± 0.7	*p* = 0.001

SE, standard error.

The SNR was significantly lower for the MATCH images and the equivalent multi-sequence images: hyper-T1 images compared with MPRAGE (3.8 ± 0.3 vs. 12.8 ± 0.6; *p* < 0.001), gray-blood compared with TOF (9.2 ± 0.6 vs. 11.9 ± 0.7; *p* = 0.007), and T2w MATCH images compared with post-contrast T1w TSE (8.9 ± 0.6 vs. 12.5 ± 0.7; *p* = 0.001). The summary of the results is presented in Table 3.

In addition, IPH on MATCH showed a significantly lower mean CNR than that on MPRAGE (10.4 ± 1.1 vs. 13.4 ± 1.4; *p* = 0.02).

### 3.2. Interobserver agreement

The summary of the interobserver agreement is presented in Table 4. The agreement between two readers on multi-sequence images was excellent for IPH (*κ* = 0.82) and calcifications (*κ* = 0.84) and substantial for LRNC (*κ* = 0.73) detection. On MATCH images, excellent, fair, and substantial types of interobserver agreement for the detection of IPH (*κ* = 0.84), calcifications (*κ* = 0.21), and LRNC (*κ* = 0.71), respectively, were observed.

**Table 4:** Interobserver agreement for multi-sequence and MATCH images.

Plaque component	Multi-sequence kappa (*κ*)	MATCH kappa (*κ*)	Multi-sequence ICC (95% CI)	MATCH ICC (95% CI)
Total vessel wall volume	–	–	0.81 (0.67–0.89)	0.77 (0.64–0.86)
The presence and total volume of LRNC	0.73	0.71	0.96 (0.93–0.98)	0.94 (0.90–0.97)
The presence and total volume of IPH	0.82	0.84	0.97 (0.94–0.98)	0.78 (0.62–0.87)
The presence and total volume of CA	0.84	0.21	0.63 (0.36–0.79)	0.61 (0.41–0.76)
Total volume of fibrous tissue	—	—	0.68 (0.44–0.82)	0.70 (0.53–0.81)
NWI	—	—	0.86 (0.75–0.92)	0.85 (0.75–0.91)

CA, calcifications.

**Figure 2:**
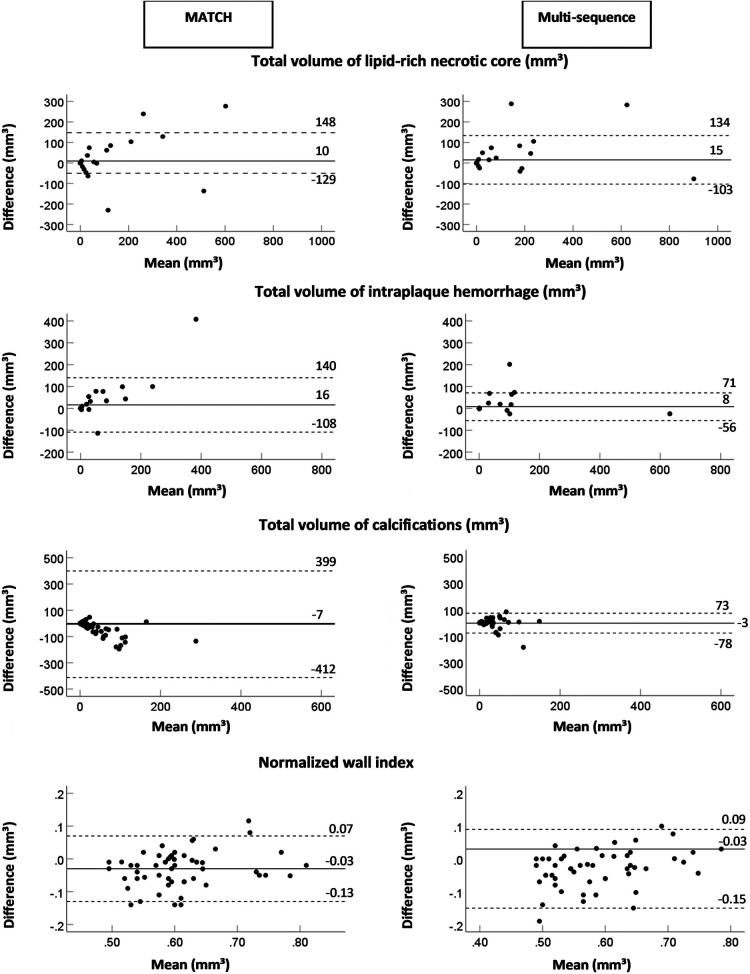
The Bland–Altman plots of the difference (mm^3^) between two readers and the mean value for the total volume of the lipid-rich necrotic core, intraplaque hemorrhage, calcifications, and the normalized wall index for MATCH and the multi-sequence protocol. The continuous lines display the mean bias, and the dashed lines display two times the SD.

The interobserver reproducibility [ICC; 95% confidence interval (CI)] of the quantification was as follows: for multi-sequence MRI, excellent for the total volume of LRNC (0.96; 0.93–0.98) and IPH (0.97; 0.94–0.98), good for the total vessel wall (0.81; 0.67–0.89) and NWI (0.86; 0.75–0.92), and moderate for fibrous tissue (0.68; 0.44–0.82) and calcifications (0.63; 0.36–0.79). When using MATCH images, interobserver reproducibility for the total volume was excellent for LRNC (0.94; 0.90–0.97); good for IPH (0.78; 0.62–0.87), total vessel wall (0.77; 0.64–0.86), fibrous tissue (0.70; 0.53–0.81), and NWI (0.85; 0.75–0.91); and moderate for calcifications (0.61; 0.41–0.76). In addition, the results of the quantitative analysis of the vessel wall and the plaque components on multi-sequence vs. MATCH images for both readers are listed in Supplementary Table S6 and shown in Figure 2.

### 3.3. The performance of MATCH for identifying and quantifying carotid plaque features

**Figure 3:**
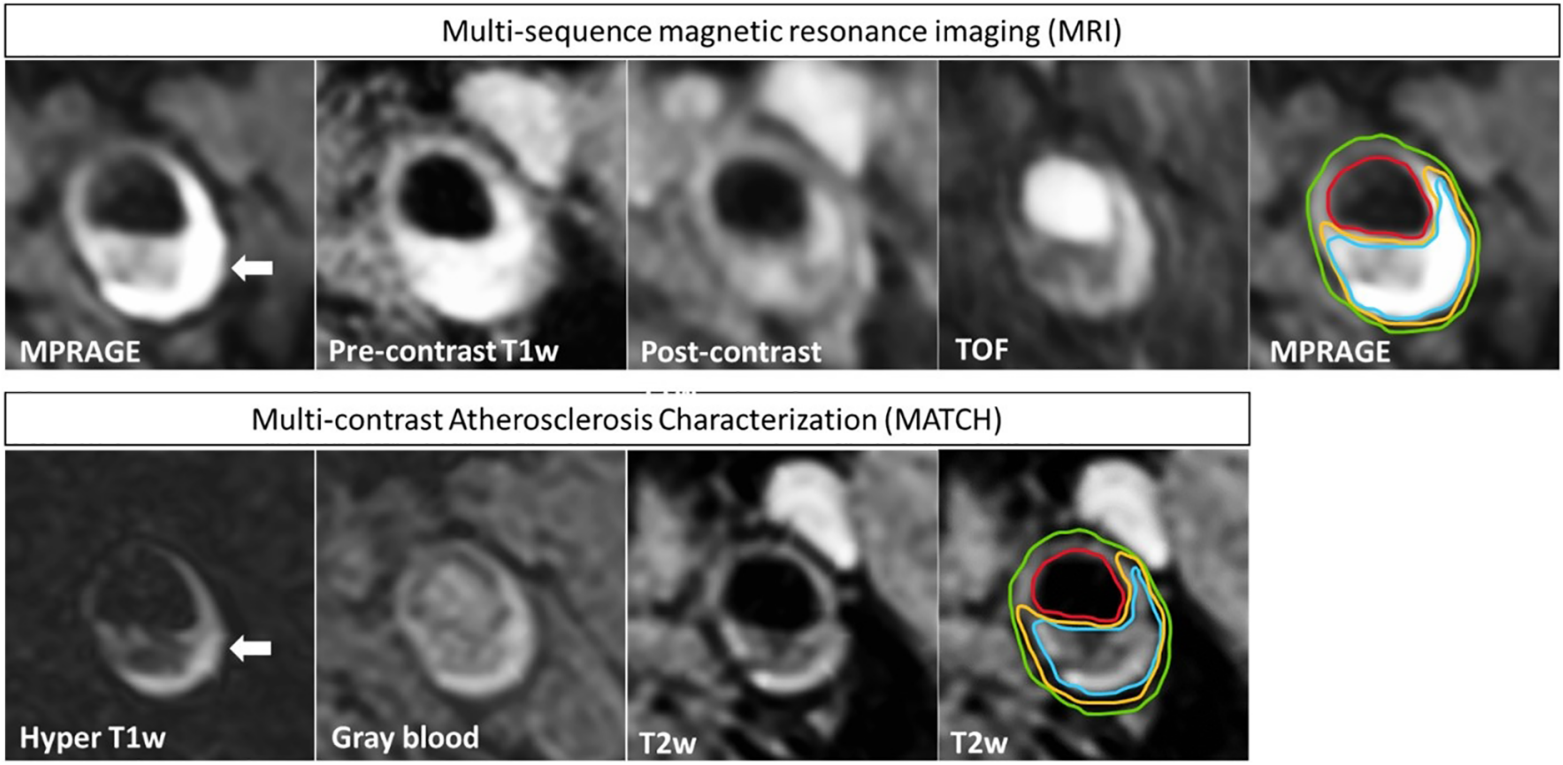
An example of a patient that exhibits an atherosclerotic plaque in the left carotid artery. The four multi-sequence MR images have been manually co-registered and are displayed in the upper row. In addition, the MATCH images with inherent co-registration are shown in the bottom row. IPH appears hyper-intense (white arrow) on hyper-T1w MATCH and MPRAGE images. Contours are shown on T2w MATCH and MPRAGE: green for the outer vessel wall, red for the lumen, yellow for LRNC, and blue for IPH. A good agreement is shown between the contours that are delineated on the MATCH vs. the multi-sequence MR images, although slight deviations can be observed.

**Figure 4:**
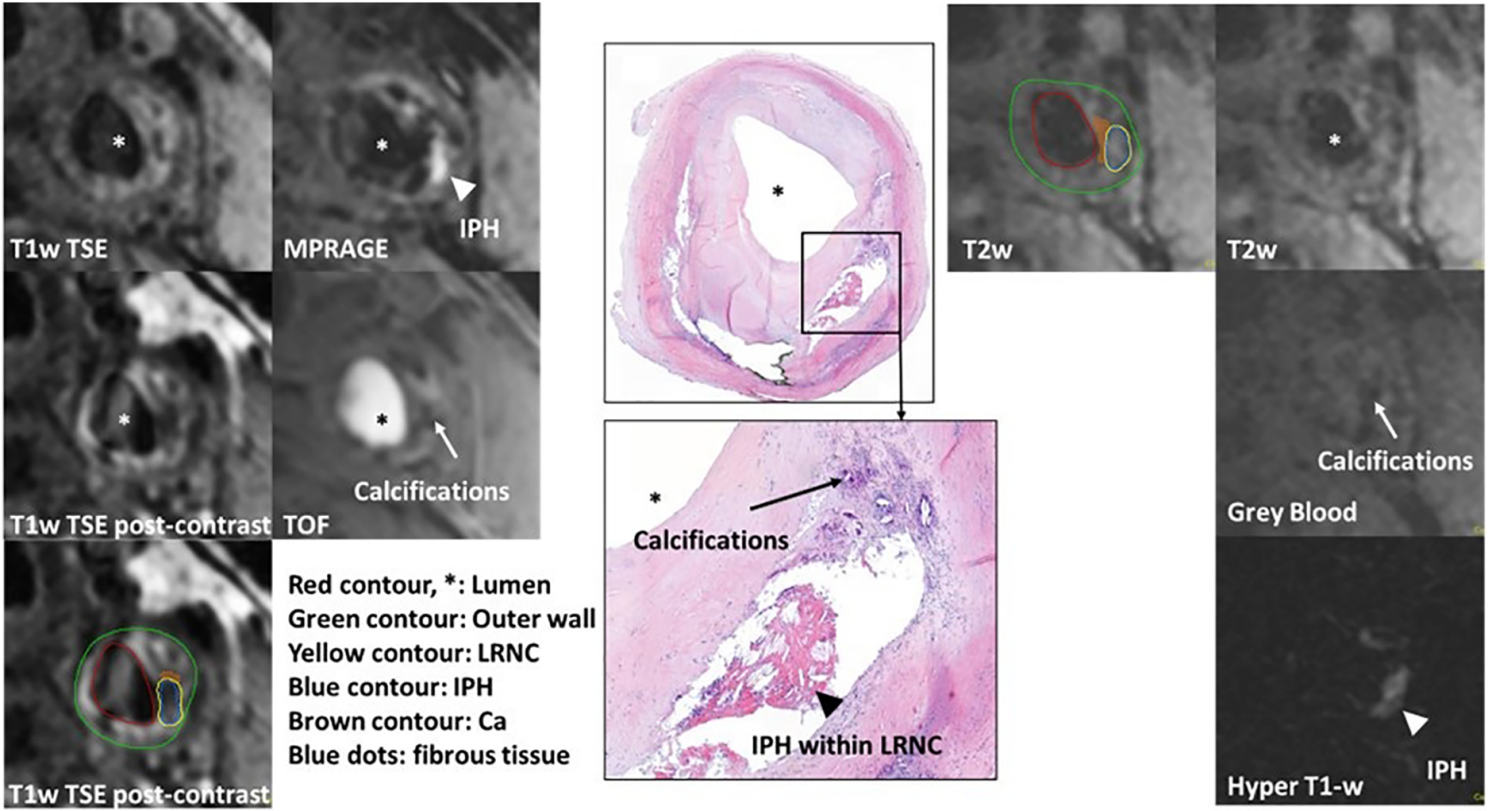
Example of histological comparison of MATCH and multi-sequence protocol at the left common carotid artery. IPH appears hyper-intense (white arrowhead) on hyper-T1w MATCH and MPRAGE images. A dark calcified nodule on the gray-blood image and TOF (white arrows). Histological specimen with hematoxylin–eosin staining confirms the presence of IPH, LRNC (black arrowhead), and calcifications (black arrows). Contours are shown on T2w MATCH and T1w post-contrast: green for the outer vessel wall, red for the lumen, yellow for the lipid-rich necrotic core, brown for calcifications, and blue for IPH.

The results of true-positive/negative and false-positive/negative identification of plaque components on MATCH images compared with multi-sequence protocol as the reference standard for both readers are listed in Table 5. For readers 1 and 2, the sensitivity and specificity in detecting IPH on hyper-T1w images and LRNC on T2w images of the MATCH protocol were ≥88.9% and 90.9% and ≥81.8% and ≥85.4%, respectively. However, lower sensitivity and specificity of 71.4% and 32.0%, respectively, were observed for scoring calcifications on the MATCH protocol gray-blood images. Figure 3 shows an example of good agreement between MATCH and multi-sequence images for identifying plaque composition. Figure 4 also shows the presence of IPH, LRNC, and calcifications on MATCH and multi-sequence images with histology as a reference in a carotid endarterectomy (CEA) specimen in a patient that underwent CEA 1 day after the MRI examination as part of routine clinical care.

**Table 5:** Concordance between multi-sequence and MATCH images.

MATCH	Multi-sequence (MPRAGE)			
			IPH−	IPH+
	IPH−	Reader 1	41 (95.3%)	1
		Reader 2	40 (90.9%)	1
	IPH+	Reader 1	2	9 (90.0%)
		Reader 2	4	8 (88.9%)
MATCH	Multi-sequence (pre- and post-contract T1w)*			
			LRNC−	LRNC+
	LRNC−	Reader 1	35 (94.6%)	2
		Reader 2	35 (85.4%)	2
	LRNC+	Reader 1	2	10 (83.3%)
		Reader 2	6	9 (81.8%)
MATCH	Multi-sequence			
			CA−	CA+
	CA−	Reader 1	10 (47.6%)	8
		Reader 2	8 (32.0%)	8
	CA+	Reader 1	11	24 (75.0%)
		Reader 2	17	20 (71.4%)

Four carotids were excluded (contraindication of contrast injection).

**Figure 5:**
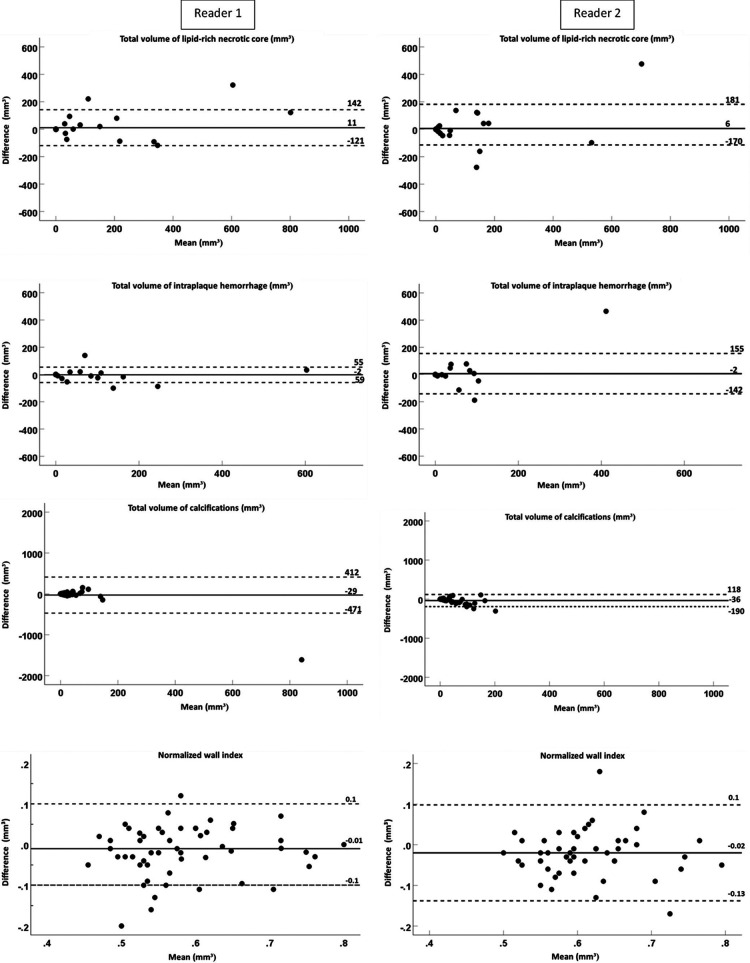
Bland–Altman plots of difference (mm^3^) between two protocols (multi-sequence and MATCH) vs. the mean value for the total volume of lipid-rich necrotic core intraplaque hemorrhage, calcifications, and normalized wall index with a mean bias (continuous lines) and two times the SD (dashed lines).

The results of the quantitative analysis of plaque components on multi-sequence and MATCH images for both readers are listed in Supplementary Table S6 and shown in Figure 5 as Bland–Altman plots. For reader 1, there was an overall significant good correlation (ICC > 0.75, *p* < 0.01) between both protocols for the quantitative plaque assessment, except for a poor correlation for the total volume of calcifications (ICC = 0.38, *p* = 0.4) and a moderate correlation for the total volume of fibrous tissue (ICC = 0.59, *p* < 0.001). For reader 2, we observed an overall good correlation for the quantification of the total vessel wall volume and total volume of LRNC, PWV, and NWI (ICC > 0.75, *p* < 0.01), a poor correlation for the total volume of calcifications (ICC = 0.37, *p* = 0.06), and a moderate correlation for the total volume of fibrous tissue (ICC = 0.70, *p* < 0.01.

No significant bias between multi-sequence MRI and MATCH was shown for the quantification of LRNC, IPH, calcifications, and NWI (*p* > 0.05) for reader 1. However, the total volumes of the vessel wall (1,335.1 ± 55.8 mm^3^ vs. 1,421.3 ± 63.2 mm^3^), fibrous tissue (1,227.8 ± 50.6 mm^3^ vs. 1,441.5 ± 68.5 mm^3^), and PWV (57.6 ± 1.3% vs. 59.5 ± 1.3%) were significantly different (*p* < 0.05) between multi-sequence and MATCH. For reader 2, no significant bias was found for quantifying LRNC, IPH, and fibrous tissue (*p* > 0.05). However, the total volumes of the vessel wall (1453.7 ± 42.8 mm^3^ vs. 1584.2 ± 65.2 mm^3^), calcifications (23.5 ± 5.7 vs. 59.6 ± 10.9), PWV (60.5 ± 1.1% vs. 62.5 ± 1.0%), and NWI (0.60 ± 0.01 vs. 0.62 ± 0.01) were significantly different (*p* < 0.05) between multi-sequence and MATCH.

## 4. Discussion

In the present study, we validated MATCH with a multi-sequence carotid MRI protocol that was recommended in a white paper (16). Our results showed a substantial-to-excellent interobserver agreement of detecting and quantifying all plaque features with MATCH except for calcification which showed a fair-to-moderate agreement. In addition, the sensitivity and specificity of identifying IPH and LRNC on the MATCH images using multi-sequence MRI as the reference standard were high. Moreover, the quantification of vulnerable carotid plaque components such as IPH and LRNC using MATCH were in agreement with the quantification using the multi-sequence protocol, whereas a moderate and poor agreement was seen for the total volume of fibrous tissue and calcifications, respectively. These findings are important since the scan time and the time needed for image analysis were substantially less for MATCH than for the conventional multi-sequence protocol.

Dai et al. (19) demonstrated that MATCH showed a comparable, if not superior, performance compared to a conventional protocol in identifying and quantifying major carotid plaque components. This previous study used the T1w TSE, T2w TSE, and TOF images as a reference. Therefore, LRNC was determined using T2w TSE images, which is less accurate than that of the contrast-enhanced T1w TSE images, which have been used in the present study (27). In addition, in the current work, we also measured additional practical outcome variables, such as the total scan time, which includes acquisition time and planning. Similarly, we compared how long it takes to identify and quantify carotid plaque features and the composition of one carotid plaque. In comparison, both MATCH and MPRAGE have similar scan times and can be used to identify IPH, an important risk factor for predicting stroke (11, 12). The advantage of MATCH is that without a time penalty, it can also provide multiple co-registered images, which can be used to identify additional plaque features, such as LRNC volume, that are not possible by using only MPRAGE. In addition, the MPRAGE sequence contains a non-selective inversion pre-pulse. This non-selective inversion pre-pulse is limited to the field of view of the body coil. Therefore, in case we observe that the luminal blood is not entirely suppressed, the patient's position can be slightly off-centered toward the foot direction. In the MATCH protocol, FSD pre-pulses are applied to suppress the signal originating from the luminal blood (17, 18). The FSD prepared is flow-dependent but does not rely on the inflow of blood (18).

We observed a poor agreement on the quantification of calcifications. The visualization of small calcifications on MR images can be challenging due to their dark appearance. Therefore, partial volume effects can obscure small regions of calcifications on MRI. In line with this, a higher measurement error for calcifications compared to that for the LRNC and wall volume was reported in a previous study on quantitative assessment of carotid atherosclerosis (28). In the multi-sequence MRI protocol, regions that were not identified as LRNC and that appeared dark on at least two different weightings were identified as calcifications. In the MATCH protocol, calcifications were identified on the gray-blood image. This may explain the poor agreement on the quantification of calcification. Also, this will lead to a lower agreement in the quantification of fibrous tissue since the fibrous tissue is identified as the remaining tissue after delineating the LRNC, IPH, and calcifications. Previous studies demonstrated that IPH and LRNC were important parameters for the risk prediction of stroke (11, 29). Importantly, these parameters can be quantified with a good-to-excellent agreement. The association with stroke risk was not reported for calcifications and fibrous tissue as identified on MRI.

Despite the lower image quality observed in the hyper-T1w and T2w MATCH images, good agreement was still observed for the quantification of IPH and the LRNC, respectively. In addition, although the CNR between IPH and muscle tissue was lower for the hyper-T1w MATCH images compared to that for the MPRAGE images, the CNR remained relatively high, allowing for a clear appearance of IPH. These findings suggest that despite the lower image quality of the MATCH images, the vulnerable plaque components (IPH and LRNC) remained discernible.

MRI-based tissue quantification is accurate and reproducible (30, 31). The requirement of acquiring multiple weightings to obtain accurate measurements can lead to longer scan times. Moreover, the acquisition of different weightings increases the risk of image misregistration, resulting in the misalignment of anatomical structures and potential inaccuracies in tissue quantification. Despite these limitations, ongoing research and technological advancements aim to optimize MRI protocols to enhance the efficiency, accuracy, and reproducibility of MRI-based tissue quantification. Apart from MATCH, other novel multi-contrast MR sequences, such as simultaneous non-contrast angiography and IPH (SNAP) (32) and bright-blood and black-blood phase-sensitive inversion recovery sequence (BOOST) (33), have recently been developed. These sequences also offer the advantage of acquiring multi-contrast images using a single sequence, thereby greatly reducing scan time and providing inherent image co-registration. However, it is important to note that these novel sequences still require additional validation to ensure their reliability and accuracy in clinical practice. In addition to the advancements in MR sequences, the integration of artificial intelligence (AI) holds tremendous potential in further enhancing MR image quality and shortening analysis times. AI techniques can aid in automating image analysis tasks, enabling more efficient and streamlined processing of MR images.

A limitation of the MATCH sequence is that no criteria are available for determining the fibrous cap status on MATCH images. A thin or ruptured fibrous cap on MRI is a well-known risk factor for recurrent stroke (29, 34), and it can be easily scored on post-contrast T1w as part of the multi-sequence protocol (27, 35). Thus, if time allows, it is still beneficial to add a post-contrast dark blood T1w MRI sequence to score the fibrous cap status. This sequence, which takes 3–5 min, will require contrast injection and needs to be acquired approximately 6 min after contrast injection. This 6 min could be used to acquire a contrast-enhanced magnetic resonance angiography (MRA) to quantify the degree of stenosis. In the present study, the MATCH images were only acquired before contrast injection. Future studies could investigate whether post-contrast MATCH images could be of added value.

One of the limitations of our study is that most patients had only an intermediate or mild degree of stenosis (<70%). Symptomatic patients with high-grade carotid stenosis (70%–99%) benefit from carotid endarterectomy (36). Thus, comparing MATCH with histology as a gold standard can be considered in the future. Symptomatic patients with mild-to-moderate stenosis (<70%) are still at increased risk of recurrent ischemic events, and the usefulness of carotid endarterectomy has not yet been determined (37). Therefore, studying the risk factors of plaque components such as IPH and LRNC using a short carotid MRI protocol and inherently co-registered images is beneficial. In addition, while we made efforts to minimize bias by conducting the image evaluation with a time interval of at least 1 month, it is important to acknowledge that blinding readers to whether they delineated the plaque components on MATCH or multi-sequence MR images is inherently impossible since the observers can recognize the weightings. Last, although the sensitivity and specificity for the identification of the plaque components were similar between the two readers, reader 2 had a larger number of false-positive findings. These results suggest that although both readers were well-trained, there is still a difference in their performance.

In conclusion, MATCH can be used for identifying and quantifying carotid plaque composition except for calcifications and fibrous cap status. Inherently co-registered images and short scan and analysis times are major advantages of MATCH.

## Data Availability

The raw data supporting the conclusions of this article will be made available by the authors, without undue reservation.

## References

[1] Donkor ES. Stroke in the 21(st) century: a snapshot of the burden, epidemiology, and quality of life. Stroke Res Treat. (2018) 2018:3238165. 10.1155/2018/3238165 30598741 PMC6288566

[2] French BR Boddepalli RS Govindarajan R. Acute ischemic stroke: current status and future directions. Mo Med. (2016) 113(6):480–6. 30228538 PMC6139763

[3] Pasternak RC Criqui MH Benjamin EJ Fowkes FGR Isselbacher EM McCullough PA Atherosclerotic vascular disease conference. Circulation. (2004) 109(21):2605–12. 10.1161/01.CIR.0000128518.26834.93 15173042

[4] Virmani R Ladich ER Burke AP Kolodgie FD. Histopathology of carotid atherosclerotic disease. Neurosurgery. (2006) 59(5 Suppl 3):S219–27; discussion S3–13. 10.1227/01.NEU.0000239895.00373.E4 17053606

[5] Bentzon JF Otsuka F Virmani R Falk E. Mechanisms of plaque formation and rupture. Circ Res. (2014) 114(12):1852–66. 10.1161/CIRCRESAHA.114.302721 24902970

[6] Golledge J Greenhalgh RM Davies AH. The symptomatic carotid plaque. Stroke. (2000) 31(3):774–81. 10.1161/01.STR.31.3.774 10700518

[7] Naghavi M Libby P Falk E Casscells SW Litovsky S Rumberger J From vulnerable plaque to vulnerable patient: a call for new definitions and risk assessment strategies: part I. Circulation. (2003) 108(14):1664–72. 10.1161/01.CIR.0000087480.94275.97 14530185

[8] Saba L Potters F van der Lugt A Mallarini G. Imaging of the fibrous cap in atherosclerotic carotid plaque. Cardiovasc Intervent Radiol. (2010) 33(4):681–9. 10.1007/s00270-010-9828-8 20237780

[9] Truijman MT Kooi ME van Dijk AC de Rotte AA van der Kolk AG Liem MI Plaque at RISK (PARISK): prospective multicenter study to improve diagnosis of high-risk carotid plaques. Int J Stroke. (2014) 9(6):747–54. 10.1111/ijs.12167 24138596

[10] Kwee RM van Oostenbrugge RJ Mess WH Prins MH van der Geest RJ ter Berg JW MRI of carotid atherosclerosis to identify TIA and stroke patients who are at risk of a recurrence. J Magn Reson Imaging. (2013) 37(5):1189–94. 10.1002/jmri.23918 23166040

[11] Schindler A Schinner R Altaf N Hosseini AA Simpson RJ Esposito-Bauer L Prediction of stroke risk by detection of hemorrhage in carotid plaques: meta-analysis of individual patient data. JACC Cardiovasc Imaging. (2020) 13(2 Pt 1):395–406. 10.1016/j.jcmg.2019.03.028 31202755

[12] van Dam-Nolen DHK Truijman MTB van der Kolk AG Liem MI Schreuder F Boersma E Carotid plaque characteristics predict recurrent ischemic stroke and TIA: the PARISK (plaque at RISK) study. JACC Cardiovasc Imaging. (2022) 15(10):1715–26. 10.1016/j.jcmg.2022.04.003 36202450

[13] Kerwin WS Hatsukami T Yuan C Zhao XQ. MRI of carotid atherosclerosis. AJR Am J Roentgenol. (2013) 200(3):W304–13. 10.2214/AJR.12.8665 23436876 PMC5520985

[14] Kassem M Florea A Mottaghy FM van Oostenbrugge R Kooi ME. Magnetic resonance imaging of carotid plaques: current status and clinical perspectives. Ann Transl Med. (2020) 8(19):1266. 10.21037/atm-2020-cass-16 33178798 PMC7607136

[15] Saba L Yuan C Hatsukami TS Balu N Qiao Y DeMarco JK Carotid artery wall imaging: perspective and guidelines from the ASNR Vessel Wall Imaging Study Group and expert consensus recommendations of the American Society of Neuroradiology. AJNR Am J Neuroradiol. (2018) 39(2):E9–31. 10.3174/ajnr.A5488 29326139 PMC7410574

[16] Saba L Moody AR Saam T Kooi ME Wasserman BA Staub D Vessel wall-imaging biomarkers of carotid plaque vulnerability in stroke prevention trials: a viewpoint from the carotid imaging consensus group. JACC Cardiovasc Imaging. (2020) 13(11):2445–56. 10.1016/j.jcmg.2020.07.046 33153534

[17] Fan Z Yu W Xie Y Dong L Yang L Wang Z Multi-contrast atherosclerosis characterization (MATCH) of carotid plaque with a single 5-min scan: technical development and clinical feasibility. J Cardiovasc Magn Reson. (2014) 16:53. 10.1186/s12968-014-0053-5 25184808 PMC4222690

[18] Fan Z Sheehan J Bi X Liu X Carr J Li D. 3D noncontrast MR angiography of the distal lower extremities using flow-sensitive dephasing (FSD)-prepared balanced SSFP. Magn Reson Med. (2009) 62(6):1523–32. 10.1002/mrm.22142 19877278 PMC2841215

[19] Dai Y Lv P Lin J Luo R Liu H Ji A Comparison study between multicontrast atherosclerosis characterization (MATCH) and conventional multicontrast MRI of carotid plaque with histology validation. J Magn Reson Imaging. (2017) 45(3):764–70. 10.1002/jmri.25444 27556726

[20] Yuan C Mitsumori LM Ferguson MS Polissar NL Echelard D Ortiz G In vivo accuracy of multispectral magnetic resonance imaging for identifying lipid-rich necrotic cores and intraplaque hemorrhage in advanced human carotid plaques. Circulation. (2001) 104(17):2051–6. 10.1161/hc4201.097839 11673345

[21] Saam T Cai JM Cai YQ An NY Kampschulte A Xu D Carotid plaque composition differs between ethno-racial groups. Arterioscler Thromb Vasc Biol. (2005) 25(3):611–6. 10.1161/01.ATV.0000155965.54679.79 15653565

[22] Saam T Yuan C Chu B Takaya N Underhill H Cai J Predictors of carotid atherosclerotic plaque progression as measured by noninvasive magnetic resonance imaging. Atherosclerosis. (2007) 194(2):e34–42. 10.1016/j.atherosclerosis.2006.08.016 16978632 PMC2243074

[23] Zhao X Underhill HR Zhao Q Cai J Li F Oikawa M Discriminating carotid atherosclerotic lesion severity by luminal stenosis and plaque burden: a comparison utilizing high-resolution magnetic resonance imaging at 3.0 Tesla. Stroke. (2011) 42(2):347–53. 10.1161/STROKEAHA.110.597328 21183749 PMC5542669

[24] Yang D Liu Y Han Y Li D Wang W Li R Signal of carotid intraplaque hemorrhage on MR T1-weighted imaging: association with acute cerebral infarct. AJNR Am J Neuroradiol. (2020) 41(5):836–43. 10.3174/ajnr.A6498 32273265 PMC7228181

[25] Koo TK Li MY. A guideline of selecting and reporting intraclass correlation coefficients for reliability research. J Chiropr Med. (2016) 15(2):155–63. 10.1016/j.jcm.2016.02.012 27330520 PMC4913118

[26] Landis JR Koch GG. The measurement of observer agreement for categorical data. Biometrics. (1977) 33(1):159–74. 10.2307/2529310 843571

[27] Cai J Hatsukami TS Ferguson MS Kerwin WS Saam T Chu B In vivo quantitative measurement of intact fibrous cap and lipid-rich necrotic core size in atherosclerotic carotid plaque: comparison of high-resolution, contrast-enhanced magnetic resonance imaging and histology. Circulation. (2005) 112(22):3437–44. 10.1161/CIRCULATIONAHA.104.528174 16301346

[28] Saam T Kerwin WS Chu B Cai J Kampschulte A Hatsukami TS Sample size calculation for clinical trials using magnetic resonance imaging for the quantitative assessment of carotid atherosclerosis. J Cardiovasc Magn Reson. (2005) 7(5):799–808. 10.1080/10976640500287703 16353440

[29] Gupta A Baradaran H Schweitzer AD Kamel H Pandya A Delgado D Carotid plaque MRI and stroke risk: a systematic review and meta-analysis. Stroke. (2013) 44(11):3071–7. 10.1161/STROKEAHA.113.002551 23988640

[30] Touzé E Toussaint JF Coste J Schmitt E Bonneville F Vandermarcq P Reproducibility of high-resolution MRI for the identification and the quantification of carotid atherosclerotic plaque components: consequences for prognosis studies and therapeutic trials. Stroke. (2007) 38(6):1812–9. 10.1161/STROKEAHA.106.479139 17463311

[31] Saam T Ferguson MS Yarnykh VL Takaya N Xu D Polissar NL Quantitative evaluation of carotid plaque composition by in vivo MRI. Arterioscler Thromb Vasc Biol. (2005) 25(1):234–9. 10.1161/01.ATV.0000149867.61851.31 15528475

[32] Wang J Börnert P Zhao H Hippe DS Zhao X Balu N Simultaneous noncontrast angiography and intraplaque hemorrhage (SNAP) imaging for carotid atherosclerotic disease evaluation. Magn Reson Med. (2013) 69(2):337–45. 10.1002/mrm.24254 22442116 PMC3418400

[33] Ginami G Neji R Phinikaridou A Whitaker J Botnar RM Prieto C. Simultaneous bright- and black-blood whole-heart MRI for noncontrast enhanced coronary lumen and thrombus visualization. Magn Reson Med. (2018) 79(3):1460–72. 10.1002/mrm.26815 28722267 PMC5811778

[34] Yuan C Zhang S-x Polissar NL Echelard D Ortiz G Davis JW Identification of fibrous cap rupture with magnetic resonance imaging is highly associated with recent transient ischemic attack or stroke. Circulation. (2002) 105(2):181–5. 10.1161/hc0202.102121 11790698

[35] Kwee RM van Engelshoven JM Mess WH ter Berg JW Schreuder FH Franke CL Reproducibility of fibrous cap status assessment of carotid artery plaques by contrast-enhanced MRI. Stroke. (2009) 40(9):3017–21. 10.1161/STROKEAHA.109.555052 19556528

[36] Barnett HJM Taylor DW Haynes RB Sackett DL Peerless SJ Ferguson GG Beneficial effect of carotid endarterectomy in symptomatic patients with high-grade carotid stenosis. N Engl J Med. (1991) 325(7):445–53. 10.1056/NEJM199108153250701 1852179

[37] Endarterectomy for moderate symptomatic carotid stenosis: interim results from the MRC European carotid surgery trial. Lancet. (1996) 347(9015):1591–3. 10.1016/S0140-6736(96)91077-6 8667868

