# Supplementary material for: Quantification of carotid plaque composition with a multi-contrast atherosclerosis characterization (MATCH) MRI sequence
Source: Front Cardiovasc Med. 2023 Aug 23;10:1227495. doi: 10.3389/fcvm.2023.1227495 (PMC10481960; doi:10.3389/fcvm.2023.1227495)
Supplement: Supplementary file 1 [file Table1.docx]

Supplementary Material

Quantification of carotid plaque composition with a multi-contrast atherosclerosis characterization (MATCH) MRI sequence

**Mohamed Kassem^1,2^, Kelly Nies^1,2^, Ellen Boswijk^2,3^, Jochem van der Pol^2^, Mueez Aizaz^1,2^, Marion J J Gijbels^4,5^, Debiao Li^6^, Jan Bucerius^7^, Werner H Mess^1,8^, Joachim E Wildberger^1,2^, Robert J van Oostenbrugge^1,9^, Rik PM Moonen^2^, Zhaoyang Fan^10^, Eline Kooi^1,2*^**

^1^Cardiovascular Research Institute Maastricht (CARIM), Maastricht University Medical Center, Maastricht, The Netherlands.

^2^Department of Radiology and Nuclear Medicine, Maastricht University Medical Center, Maastricht, The Netherlands.

^3^Department of Rehabilitation Medicine, Amsterdam University Medical Center, location VUmc, Amsterdam, the Netherlands.

^4^Department of Pathology, Cardiovascular Research Institute Maastricht, Maastricht University Medical Centre, Maastricht, The Netherlands.

^5^Department of Medical Biochemistry, Experimental Vascular Biology, Amsterdam Cardiovascular Sciences, Amsterdam Infection and Immunity, Amsterdam UMC, Amsterdam, The Netherlands.

^6^Biomedical Imaging Research Institute, Cedars-Sinai Medical Center, Los Angeles, CA, USA.

^7^Georg-August University Göttingen, Department of Nuclear Medicine, Universitätsmedizin Göttingen, Göttingen, Germany.

^8^Department of Clinical Neurophysiology, Maastricht University Medical Centre, Maastricht, The Netherlands.

^9^Department of Neurology, Maastricht University Medical Centre, Maastricht, The Netherlands.

^10^Department of Radiology, University of Southern California, Los Angeles, CA, USA

*** Correspondence:** Prof. dr. M. Eline Kooi

Department of Radiology and Nuclear Medicine, Maastricht University Medical Centre, P.O. Box 5800, 6202 AZ Maastricht, The Netherlands.
eline.kooi@mumc.nl

# Supplementary Figures and Tables

## Supplementary Table 6: Comparison of MATCH and conventional multi-sequence protocol in quantifying plaque components.

| **Parameter** | **Reader** | **Protocol** | **Mean ± SE** | **95% CI of difference** | **P-value** | **ICC (95% CI)** | **P-value** |
| --- | --- | --- | --- | --- | --- | --- | --- |
| Total vessel wall volume (mm³) | 1 | Multi-sequence | 1335.1 ± 55.8 | (-140.6)-(-31.6) | 0.003 | 0.93 (0.89-0.96) | <0.01 |
|  |  | MATCH | 1421.3 ± 63.2 |  |  |  |  |
|  | 2 | Multi-sequence | 1453.7 ± 42.8 | (-230.0)-(31.0) | 0.01 | 0.76 (0.58-0.86) | <0.01 |
|  |  | MATCH | 1584.2 ± 65.2 |  |  |  |  |
| Total LRNC volume (mm³) | 1 | Multi-sequence | 67.8 ± 24.9 | (-8.5)-(29.9) | 0.27 | 0.95 (0.92-0.97) | <0.01 |
|  |  | MATCH | 57.1 ± 20.9 |  |  |  |  |
|  | 2 | Multi-sequence | 50.5 ± 21.4 | (-19.8)-(31.2) | 0.7 | 0.88 (0.79-0.93) | <0.01 |
|  |  | MATCH | 44.8 ± 15.7 |  |  |  |  |
| Total IPH volume (mm³) | 1 | Multi-sequence | 30.1 ± 13.9 | (-10.1)-(5.8) | 0.60 | 0.97 (0.96-0.99) | <0.01 |
|  |  | MATCH | 32.2 ± 14.1 |  |  |  |  |
|  | 2 | Multi-sequence | 24.0 ± 13.5 | (-15.3)-(27.0) | 0.6 | 0.84 (0.77-0.90) | <0.01 |
|  |  | MATCH | 17.5 ± 6.1 |  |  |  |  |
| Total calcifications volume (mm³) | 1 | Multi-sequence | 24.1 ± 5.5 | (-91.2)-(32.9) | 0.35 | 0.38 (0.23-0.46) | 0.4 |
|  |  | MATCH | 53.2 ± 33.6 |  |  |  |  |
|  | 2 | Multi-sequence | 23.5 ± 5.7 | (-58.5)-(-13.7) | <0.01 | 0.37 (-0.1-0.64) | 0.06 |
|  |  | MATCH | 59.6 ± 10.9 |  |  |  |  |
| Total fibrous tissue volume (mm³) | 1 | Multi-sequence | 1227.8 ± 50.6 | (-336.6)-(-90.6) | 0.001 | 0.59 (0.29-0.76) | <0.01 |
|  |  | MATCH | 1441.5 ± 68.5 |  |  |  |  |
|  | 2 | Multi-sequence | 1369.6±39.7 | (-161.8)-(33.8) | 0.2 | 0.70 (0.48-0.83) | <0.01 |
|  |  | MATCH | 1433.6±52.5 |  |  |  |  |
| Percent wall volume (PWV) % | 1 | Multi-sequence | 57.6 ± 1.3 | (-3.7)-(-0.2) | 0.03 | 0.85 (0.74-0.91) | <0.01 |
|  |  | MATCH | 59.5 ± 1.3 |  |  |  |  |
|  | 2 | Multi-sequence | 60.5 ± 1.1 | (-3.4)-(-0.6) | <0.01 | 0.87 (0.78-0.93) | <0.01 |
|  |  | MATCH | 62.5 ± 1.0 |  |  |  |  |
| Normalized wall index (NWI) | 1 | Multi-sequence | 0.58 ± 0.01 | (-0.5)-(0.0) | 0.06 | 0.85 (0.74-0.91) | <0.01 |
|  |  | MATCH | 0.60 ± 0.01 |  |  |  |  |
|  | 2 | Multi-sequence | 0.60 ± 0.01 | (-0.04)-(-0.0) | 0.01 | 0.82 (0.68-0.90) | <0.01 |
|  |  | MATCH | 0.62 ± 0.01 |  |  |  |  |
